# The Era of Precision Nutrition in the Field of Reproductive Health and Pregnancy

**DOI:** 10.3390/nu15143128

**Published:** 2023-07-13

**Authors:** Fatima Ahmad, Cinzia Myriam Calabrese, Annalisa Terranegra

**Affiliations:** 1 Division of Biological and Biomedical Sciences (BBS), College of Health & Life Sciences (CHLS), Hamad Bin Khalifa University (HBKU), Doha 34110, Qatar; faah40638@hbku.edu.qa; 2 Sidra Medicine, Translational Medicine Department, Doha 26999, Qatar; 3 BioMedLab, Politecnico di Torino, 10129 Torino, Italy; cinzia.calabrese@gmail.com

When it comes to reproductive health, various lifestyle habits can act as major contributors to either an optimized or worsened scenario of female and male fertility. “Let food be thy medicine, and let medicine be thy food”. The aforementioned notion is true in every sense. Applied to both men and women, it is becoming ever more clear that fertility and pregnancy outcomes can be strongly predicted and altered through different dietary types and components. Maintaining a healthy diet can help optimize reproductive functions and increase the chances of conceiving by affecting hormonal balance, ovulation and menstruation, sperm quality and quality, as well as inflammation and oxidative stress [1]. Poor nutrition plays a negative role in shaping the health and development of both the mother and the baby, as it can induce various metabolic disorders, including obesity and gestational diabetes mellitus (GDM) [2]. The latter is thought to be highly associated with an increased risk of preterm birth, preeclampsia and neurodevelopmental as well as cognitive disorders in offspring [3].

Previous studies have shed light on the potential impact of preconception dietary interventions to boost fertility [4]. Interestingly, two papers in this Special Issue highlight the importance of “fertility diet” and dietary components in managing female and male infertility. First, the study by Fabozzi G et al. [5] explored the possible ways to manage female infertility through “precision and personalized nutrition” according to different parameters: (1) nutrigenetics (folate intake and *MTHFR* gene), (2) microbiomics (gluten-free foods and dominance of *Lactobacillus* and *Bifidobacterium* strains), (3) metabolomics (Zonulin and increased intestinal permeability) and (4) nutrigenomics (epigenetic anti-inflammatory property of curcumin). The authors discussed the importance of the management of chronic low-grade inflammation or pro-inflammatory status associated with female infertility or negatively linked with different reproductive processes including ovulation, implantation, placentation, and conception. They argued that a combination of dietary changes (adhering to the Mediterranean diet and consuming whole grains, fruits, vegetables, fish, etc.) and supplements (vitamin B12, vitamin D, folates zinc, and omega 3), targeted as an anti-inflammatory approach to improve fertility outcomes, should be further investigated in the field of “personalized nutrition”.

Moreover, in this Special Issue, and in addition to exploring nutrition and female infertility, Ostojic SM et al. [6] reviewed the advantages of incorporating creatine, a naturally occurring and conditionally essential nutrient, into the diet of prospective fathers, as a paternal preconception dietary supplement. Creatine, known for its role in energy metabolism, has been found to influence various aspects of male reproductive health, such as sperm quality, motility, and ATP production in spermatozoa. Therefore, the authors proposed that including creatine in the preconception diet of prospective fathers could be a potential strategy for enhancing sperm viability and optimizing reproductive outcomes.

The issue of misinformation in the area of nutrition during pregnancy and later after delivery is rising nowadays, especially due to the uncontrollable spread of unscientific information via social media. The article by Verduci E et al. [7] underscored the significance of countering false information on pregnancy-related materials with trustworthy sources by introducing Nutripedia or “Nutripedia-Informati per Crescere” as a parent-oriented project and an online platform (a website, a Facebook page, and an app for IOS and Android) to educate pregnant women and to share scientific-based nutritional knowledge, especially during preconception, pregnancy and early childhood up to three years of age. They explained how inappropriate dietary choices can have adverse effects on both mothers’ and infants’ health.

To determine and assess the impact of GDM history and overweight or obesity on the dietary quality and physical activity of pregnant women, Muhli E et al. [8] conducted a cross-sectional study on 1034 pregnant women in their early to mid-gestation period in Finland by evaluating their dietary quality and physical activity through using the Index of Diet Quality (IDQ) and metabolic equivalent (MET) index, respectively. The results showed no significant differences between women with and without a history of GDM in terms of dietary quality and physical activity, but the difference was exhibited in women with overweight or obesity who were assessed with lower dietary quality and physical activity levels in comparison to those with a normal BMI. So, targeted dietary and physical activity interventions and counseling for pregnant women with a history of GDM and those with overweight or obesity are substantial to improve maternal and fetal health outcomes [8].

One of the challenging events facing mothers of preterm infants is related to nutritional care and measures that are crucial to ensure proper development and growth of their bodies. A paper published in this Special Issue by Chetta KE et al. [9] investigated the natural formation, significance and potentially harmful effects of cytotoxic α-lactalbumin-oleic acid complex named “HAMLET,” (Human Alpha-Lactalbumin Made Lethal to Tumor cells) in the human milk diet of preterm birth offspring. These protein–lipid complexes have been observed to exhibit cytotoxic effects and induce inflammatory pathways in intestinal and immune cells of preterm infants. They emphasized the need for further neonatal research to understand the specific mechanisms (e.g., analysis of the stool or milk microbiome) and long-term impacts of these cytotoxic complexes on gut permeability and inflammation.

Another paper in this Issue focused on the significant influence of the maternal diet and the weaning process on infants’ growth. Guzzardi MA et al. [10] used mice as an animal model to show how the visceral network, consisting of the gut, adipose tissue, and liver, matures in response to the weaning process and adult age. Their research work also examined the exacerbation in the weaning reaction, by enhancing the organs’ maturation process and liver inflammation upon the exposure to maternal high-fat diet. During weaning, changes in microbiota composition were reported with a dominance of *Clostridia* and *Bacteroidia* classes, higher computerized tomography (CT) density reflecting inflammatory cascades and overexpression of metabolic pathways within the visceral network involved in constitutive growth, sphingolipid metabolism and inflammation [10]. The authors recommended future studies to assess CT radiodensity and glucose uptake in the visceral network along with microbiota composition in order to investigate its function in the weaning reaction.

In summary, the studies published in this Special Issue, in addition to all research studies investigating the complex relationship between nutrition and reproductive health, are essential to advance our knowledge and understanding in this area. These studies provided new insights into how dietary choices and dietary intake can affect male and female fertility (ovulatory disorders, hormonal imbalance and sperm quality), pregnancy complications (GDM and preterm birth) and infant development (cognitive and immune functions, metabolic disorders). This knowledge will encourage healthcare providers to create new strategies to improve fertility and pregnancy outcomes by adhering to evidence-based dietary interventions (offering appropriate dietary advice, supplementation strategies, and lifestyle modifications) and personalized nutrition approaches.
